# Precision Medicine: IL-1RA and Pancreatic Cancer Organoids

**DOI:** 10.3390/biology14060604

**Published:** 2025-05-25

**Authors:** Annah G. Morgan, Michelle F. Griffin, Michael T. Longaker, Jeffrey A. Norton

**Affiliations:** 1 Hagey Laboratory for Pediatric Regenerative Medicine, Division of Plastic and Reconstructive Surgery, Department of Surgery, Stanford University School of Medicine, Stanford, CA 94305, USA; agmorgan@stanford.edu (A.G.M.); mgriff12@stanford.edu (M.F.G.); longaker@stanford.edu (M.T.L.); 2 Division of Plastic and Reconstructive Surgery, Department of Surgery, Stanford University School of Medicine, Stanford, CA 94305, USA; 3 Division of General Surgery, Department of Surgery, Stanford University School of Medicine, Stanford, CA 94305, USA

**Keywords:** organoids, precision medicine, air–liquid interface (ALI) culture, pancreatic ductal adenocarcinoma (PDAC), cancer-associated fibroblasts (CAFs)

## Abstract

Cancer organoids have emerged as transformative models for studying tumor biology and therapeutic responses due to the ability to replicate the complexity of the tumor microenvironment (TME). Tumor organoids recapitulate the genetic and phenotypic diversity of cancers, making them invaluable for investigating mechanisms of resistance and identifying novel therapeutic targets. Patient-derived organoids (PDOs) allow specific treatment methods to be designed based on the properties of each individual tumor in vitro. Pancreatic ductal adenocarcinoma (PDAC) is an aggressive cancer with an immunosuppressive nature. PDAC has a poor prognosis, with the survival rates of metastatic PDAC being improved only minimally over the last few decades. In this study, we demonstrate the antitumor effects of an IL-1 receptor antagonist (IL-1RA) in murine and human PDAC organoids. By reducing the burden of suppressive tumor elements like CAFs, IL-1RA treatment facilitates better immune cell access and response.

## Simple Summary

Organoids are three-dimensional clusters of cells grown in culture that mimic the structure and function of organs in the body. Organoid models can help scientists study how diseases like cancer develop and how they respond to treatments, as well as provide a more accurate way to test new therapies and understand disease behavior without testing on patients. This study focuses on the use of cancer organoids. Specifically, it explores pancreatic ductal adenocarcinoma, a type of cancer with a very poor prognosis. In this study, IL-1 receptor antagonist (IL-1RA) was used to treat these pancreatic cancer organoids. The results showed that IL-1RA had antitumor effects in both human and mouse models of pancreatic cancer. The findings suggest that this treatment could offer a new way to fight pancreatic cancer and possibly improve the effectiveness of treatments for other cancers as well. Using organoids generated from a patient’s diseased tissue to test and develop treatments could lead to more-personalized treatment options, benefiting patients by offering effective, targeted therapies.

## 1. Introduction

Pancreatic ductal adenocarcinoma (PDAC) is a highly lethal malignancy with limited treatment options [1,2]. Currently, the only potentially curative treatment is complete surgical resection. Pancreatic tumors entangle themselves into surrounding tissue and vasculature, making surgical extirpation challenging. Even in patients with successful surgery, the 5-year survival rate is only approximately 10–20% [1,3,4]. As most patients present with locally advanced disease, neoadjuvant chemotherapy and radiation therapy have gained enthusiastic acceptance to try to make tumors more resectable [2,5,6,7,8,9,10]. However, neoadjuvant therapy requires that the surgeon and patient must delay potentially effective surgery. Furthermore, in some instances chemotherapy and radiation therapy may be totally ineffective. Currently, 5-FU and gemcitabine chemotherapy are established drugs for PDAC treatment, but other treatments need to be developed. New approaches and targets are needed [5]. PDAC exhibits a highly immunosuppressive tumor microenvironment (TME), composed predominantly of stromal cells, tumor cells, immune cells, and extracellular matrix (ECM) components [1,7]. Cancer-associated fibroblasts (CAFs) have recently become a target of interest in the treatment of solid tumors. CAFs are a heterogeneous population of stromal cells from which many subtypes have been identified [1]. CAFs are the most abundant cell type in the TME [1]. They are responsible for secreting the extracellular matrix that composes a majority of the dense tumor stroma, inhibiting immune recognition of the tumor, and supporting tumor metabolism by promoting a hypoxic environment [1]. Developing therapies to target CAFs is a promising strategy for cancer treatment.

Precision medicine, or precision oncology, is a growing field of research. Precision oncology is a specialized approach to cancer treatment that utilizes the molecular profiling of individual tumors to identify specific genetic alterations and then customize treatment plans accordingly [11]. The goal of precision oncology is to develop therapies that are more effective and less toxic than standard therapies by focusing on the unique characteristics of each patient’s cancer. This practice is becoming more popular as bioinformatics technology becomes more advanced. However, there is still room for improvement, particularly with next-generation sequencing. Next-generation sequencing (NGS) is sequencing technology that allows for the rapid and cost-effective sequencing of DNA and RNA [12]. While this technology is revolutionary for the advancement of precision medicine, there is still crucial information that can be missed. The readout is dependent on the size of the tumor sample, the areas of genes analyzed, and each lab’s technical validity standards [11,12]. One way to test the applicability of these results on a tumor without testing on a patient would be to utilize patient-derived organoids.

Patient-derived organoids (PDOs) are a novel method for developing personalized treatment plans. A patient’s tumor is used to generate an organoid that can model the intricacies of the TME. These PDOs can be used to identify drug sensitivity and the unique genetic characteristics of individual tumors [13]. There are many methods by which organoids can be developed. In this study, we use the air–liquid interface (ALI) culture method, a 3D culture system which facilitates generation of organoids that closely resemble the tumor microenvironment with conservation of native stromal [14] and immune components [15,16]. In ALI culture, tumor fragments are embedded in a collagen gel matrix in which one surface is in contact with the liquid culture medium and the other is exposed to air [15,16]. ALI culture establishes a tumor milieu that is most like the true TME [17]. Experimental treatment can be applied to the tumor organoid and tumor viability can then be assessed. Treatments that prove to be effective in organoids can subsequently be used for the treatment of patients. This method can be used to identify the precise effective treatment for each individual patient’s tumor in vitro which can then be administer in vivo [5,6,7,8,9,10,11,12,13,14,15,16].

In this paper, we provide methods for the establishment of organoids in the laboratory, and we report initial results of organoid treatment using interleukin 1 receptor antagonist (IL-1RA) in murine and human PDAC organoids [2,16,18,19,20]. IL-1RA has been used as an anti-CAF treatment for other solid cancers, such as colon cancer [21,22], but its effects on CAFs in PDAC have not been thoroughly investigated.

## 2. Materials and Methods

### 2.1. Mice and Humans

This study utilized male and female B6 (C57BL/6J) mice (*n* = 3) aged between 6 and 8 weeks (acquired from Jackson Laboratories). Following approved protocols by Stanford’s Laboratory Animal Care (APLAC #34166), mice were accommodated in the Stanford University Comparative Medicine Pavilion and were under the care and monitoring of the Veterinary Service Center. Human tumor samples were obtained from patients undergoing PDAC resection procedures (*n* = 3) following an approved protocol by Stanford’s Institutional Review Board, #45051. The human samples came from three patients, two male and one female. These patients were between 65 and 73 years of age. All patients had resectable tumors, localized to the pancreas (stages 1 and 2), and underwent Whipple procedures.

### 2.2. Tissue Collection and Preparation

For mouse tumor organoids, cells isolated from the LSL-KrasG12D; LSL-Trp53R172H; and Pdx1-Cre mouse (KPC cells) (Cat#153600; CancerTools, Stratford Cross, London, UK) were implanted into the pancreas of C57BL/6J mice to induce the formation of PDAC tumors. Cells were implanted into the tail of the pancreas after ligation of the splenic artery and vein. Occlusion of the splenic blood flow induces pancreatitis, but not ischemia, because of collateral blood flow via the short gastric vessels. Tumors were allowed to develop over the course of two weeks. For human tumor organoids, PDAC tissue was obtained from Whipple procedures performed at Stanford Hospital. Approximately 100 mg of PDAC tumor tissue was collected from mice or human patients. After collection, the tissue was immediately submerged in DMEM containing 1% antibiotic-antimycotic and kept hydrated until ready for use. The tissue was then minced into a paste-like consistency and washed with additional DMEM with 1% antibiotic-antimycotic to eliminate any fat from the tissue. The mixture was centrifuged at 500 rcf for 5 min at 4° Celsius and the supernatant was discarded. This process was repeated if the tissue had excess fat.

### 2.3. Preparation of Collagen Gel

The collagen gel matrix comprised rat tail collagen type 1 (Cat#634-00663; Nitta Gelatin Inc., Morrisville, NC, USA), 0.2M HEPES buffer (Cat#J60712-AP; ThermoFisher Scientific, Waltham, MA, USA), and 10× MEM (Cat#11430030; ThermoFisher Scientific). The pH of the HEPES buffer was adjusted using small volumes of NaOH and HCl to reach a pH of 9.0. To make a single gel, 400 μL of rat tail collagen type 1 was first combined with 50 μL of the 9.0 pH HEPES buffer. Once thoroughly mixed, 50 μL of 10× MEM was added to the solution. With the addition of 10× MEM the solution turns pink, and once fully mixed should be a pale orange. Components should be combined in this exact order. The gel mixture should be kept on ice until ready for use.

### 2.4. Organoid Culture

Minced, washed tumor tissue was resuspended in the collagen gel mixture. Then, 200 μL of the tissue–gel mixture was added into a 0.4 μm transwell insert (Cat#CLS3470-48EA; Corning, Corning, NY, USA) in a 24-well plate. The gels were incubated at 37° Celsius for one hour to set. Once solid, 500 μL of the prepared organoid medium was added to each well, outside of the transwell insert. The medium was aspirated and replaced the following day, as well as every other day thereafter. Organoids may take 1–2 weeks to form [23,24]. Organoids were treated with 10 μg/mL of an IL-1 receptor antagonist (Cat#AF12198; MedChemExpress, Monmouth Junction, NJ, USA) daily for 5 days beginning on post-implantation day 7.

The base organoid medium comprised RMPI-1640 medium (Cat#11875093; Gibco, Waltham, MA) supplemented with 1% (5 mL) antibiotic-antimycotic (Cat#A5955; Sigma-Aldrich, St. Louis, MO, USA) and 10% (50 mL) fetal bovine serum (Cat#A5670701; Gibco). For mouse tumor organoids the base medium was supplemented with 500 IU/mL murine recombinant IL-2 protein (Cat# 212-12-20UG; Peprotech, Cranbury, NJ, USA), 50% *v*/*v* Wnt-3a conditioned medium (Cat# SCM112; Sigma-Aldrich), 50 ng/mL Mouse Epidermal Growth Factor (Cat# 2028-EG-200; Bio-techne, Minneapolis, MN, USA), 100 ng/mL recombinant murine Noggin (Cat# 50688-M02H; SinoBiological, Beijing, China), and 10% *v*/*v* R-spondin1 conditioned medium (Cat# SCM104; Sigma-Aldrich). For human tumor organoids, the base medium was supplemented with 500 IU/mL human recombinant IL-2 protein (Cat#589104; Biolegend, San Diego, CA, USA), 50% *v*/*v* Wnt-3a conditioned medium (Cat# SCM112; Sigma-Aldrich), 50 ng/mL human Epidermal Growth Factor (Cat#78006.1; StemCell Technologies, Vancouver, Canada), 100 ng/mL recombinant human Noggin (Cat# 78060.1; StemCell Technologies), and 10% *v*/*v* R-spondin1 conditioned medium (Cat# SCM104; Sigma-Aldrich) [25,26].

### 2.5. Immunofluorescence

Organoids in the collagen gel were fixed in 4% paraformaldehyde (Cat# J61899-AK; ThermoFisher Scientific) for one hour. Organoid gels were washed 2× with PBS for 15 min. Organoids were permeabilized with 0.1% triton for 10 min followed by 1 h in 1× Power Block™ (Cat#HK085-GP; Biogenex, Fremont, CA). After blocking, primary antibodies were applied at a 1:100 concentration of antibody to Power Block™ for 1 h. The following primary antibodies were used: Anti-collagen 1 (Cat#MA1-26771; ThermoFisher Scientific), Anti-alpha smooth muscle actin (Cat#ab7817; Abcam, Cambridge, UK), Anti-Focal Adhesion Kinase (Cat# AHO1272; ThermoFisher Scientific), Anti-CD4 (Cat#RM1013; Abcam), Anti-CD8 alpha (Cat#RM1129; Abcam, Anti-CXCL12 (Cat# MA5-23759; ThermoFisher Scientific), Anti-EpCAM (Cat# BS-0593R; ThermoFisher Scientific), Anti-VEGF (Cat# MA5-13182; ThermoFisher Scientific), and Anti-MHC II (Cat# ab55152; Abcam). Following a series of washes, organoids were covered and incubated for 2 h with Alexa Fluor™ Plus 488 (Cat#A32723; ThermoFisher Scientific) or Alexa Fluor™ Plus 594 (Cat#A32731; ThermoFisher Scientific) secondary antibodies at a 1:200 concentration of antibody to Power Block™. Organoid gels were mounted in Fluoromount-G mounting solution with DAPI (Cat#00-4959-52; ThermoFisher Scientific). Fluorescent images were acquired with a Zeiss LSM880 inverted confocal, Airyscan, AiryscanFAST, and GaAsP detector upright confocal microscope.

### 2.6. Statistical Analysis

Statistical analysis was performed using GraphPad Prism v10. Unpaired *t*-tests were used to compare two groups. Statistical significance was considered at *p* < 0.05 for all tests. Pixel density calculation and measurements were performed using ImageJ Version 2.

## 3. Results

### 3.1. Murine PDAC Organoids

**Figure 1:**
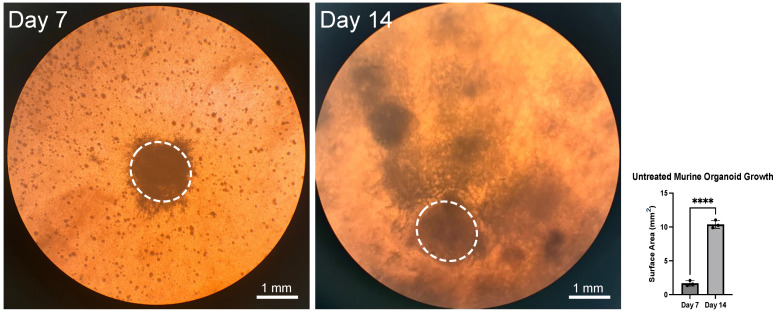
Murine pancreatic ductal adenocarcinoma (PDAC) organoid. The gross image shows the same organoid at day 7 (**left**) and day 14 (**right**) after implantation. By day 14 the organoid has significantly increased in size (*p* < 0.0001). The dotted lines outline the original tumor fragment that was implanted. Four asterisks (****) indicate that the *p* value is less than 0.0001.

We first assessed gross morphology. We found that we had the best result generating organoids from freshly dissected PDAC. Tumor fragments were seeded into the collagen gel matrix, and by day 7 we saw a larger tumor fragment (approximately 1 mm in diameter) surrounded by smaller spheroid structures. The border of the large fragment is not smooth, as the tumor appears to be invading into the adjacent collagen matrix. By day 14 the tumor had grown significantly. There is evidence of direct invasion into contiguous gel in multiple areas (Figure 1).

**Figure 2:**
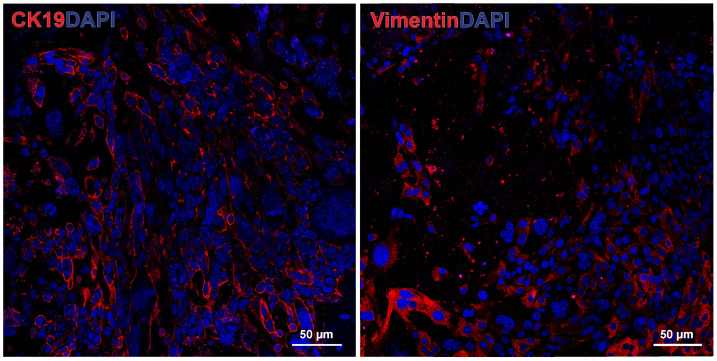
Murine pancreatic ductal adenocarcinoma (PDAC) organoid stained with cytokeratin 19 (CK19), an epithelial cell marker [29] (**left**), and vimentin, a fibroblast marker (**right**).

To verify that two major cell types of interest (epithelial tumor cells and cancer-associated fibroblasts) were preserved, we performed immunofluorescence staining for the markers cytokeratin 19 (CK19), a marker of keratin, normally expressed by epithelial cells in the lining of the gastroenteropancreatic tract, and vimentin, a marker of mesenchymal cells such as fibroblasts. The untreated murine PDAC tumor organoid showed substantial CK19 staining, representing the epithelial pancreatic tumor cells, and vimentin staining, representing the cancer-associated fibroblasts [17,27,28] (Figure 2).

**Figure 3:**
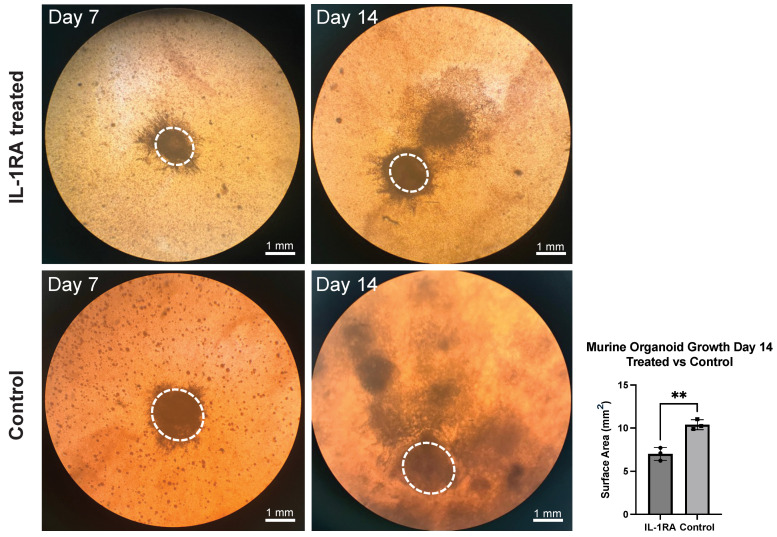
Murine pancreatic ductal adenocarcinoma (PDAC) organoid at post-implantation days 7 and 14. The dotted lines outline the original tumor fragment that was implanted. The untreated control tumor organoid (**bottom row**) is compared to the IL-1 receptor antagonist (IL-1RA)-treated tumor organoid (**top row**). IL-1RA treatment decreases the tumor organoid size significantly (*p* < 0.005). Two asterisks (**) indicate that the *p* value is less than or equal to 0.01, but greater than 0.001.

Performing preliminary studies with pancreatic adenocarcinoma in mice and humans, we found that IL-1 signaling is upregulated in PDAC compared to a normal pancreas. Treating murine PDAC tumors with IL-1RA in vivo decreases tumor size as well as fibrosis. The IL-1 receptor antagonist (IL-1RA) blocks the development of tumor inflammation [30]. In murine PDAC organoids, IL-1RA treatment decreases the magnitude of growth (top right) compared to the untreated control tumor organoid (lower right) (Figure 3).

**Figure 4:**
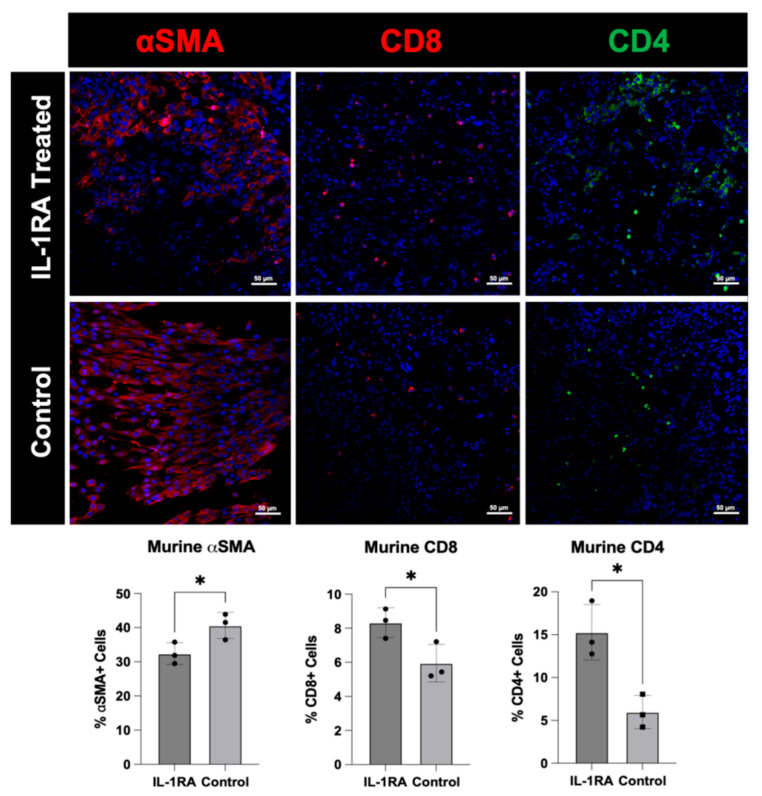
Immunofluorescence staining of murine pancreatic ductal adenocarcinoma (PDAC) organoids harvested on day 14. The tumor organoids were either untreated control (**bottom row**) or treated with IL-1RA (**top row**). IL-1RA treatment significantly decreased expression of alpha-smooth muscle actin (α-SMA) (*p* < 0.05), and significantly increased the expression of CD4+ (*p* < 0.05) and CD8+ (*p* < 0.05) immune cells. The single asterisk (*) indicates that the *p* value is less than or equal to 0.05, but greater than 0.01.

Immunofluorescence staining was performed on the murine PDAC organoids harvested on day 14 using an antibody to alpha-smooth muscle actin (α-SMA), a marker of smooth muscle cells and a population of CAFs known as myofibrotic or myofibroblastic CAFs (myCAFs) [1]. MyCAFs are primarily responsible for secreting the dense extracellular matrix proteins in the tumor [1]. The organoids were also stained with antibodies to CD8 and CD4, primarily used to identify T helper cells and cytotoxic T cells respectively, but can also be expressed by other immune cell populations [7]. The staining reveals that IL-1RA treatment decreases the number and density of α-SMA expressing cells. The expression of both CD8 and CD4 is increased with IL-1RA treatment, suggesting that there is an increased immune response following the inhibition of IL-1 signaling [30,31] (Figure 4).

**Figure 5:**
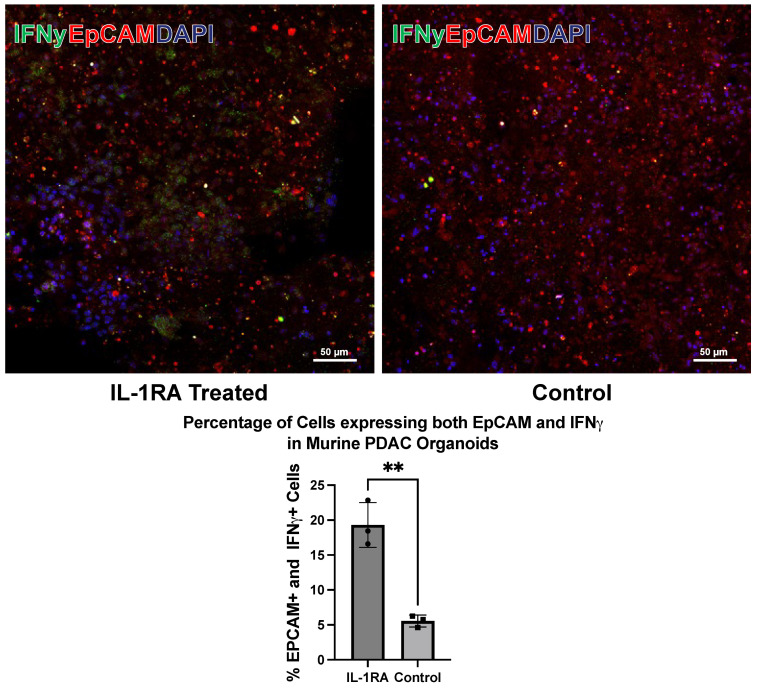
Immunofluorescence staining of murine pancreatic ductal adenocarcinoma (PDAC) tumor organoid harvested on day 14 with antibodies to interferon gamma (IFNγ), a cytokine implicated in tumor cell death, and epithelial cell adhesion molecule (EpCAM), a marker of epithelial tumor cells. The images show that, with IL-1RA treatment, the amount of EpCAM-expressing tumor cells that also express IFNγ increases significantly (*p* < 0.005). Two asterisks (**) indicate that the *p* value is less than or equal to 0.01, but greater than 0.001.

To assess tumor cell death in response to IL-1RA treatment, tumor organoids were stained with antibodies to epithelial cell adhesion molecule (EpCAM) (red), a transmembrane protein expressed on the surface of epithelial cells, and interferon-γ (IFN-γ) (green). Interferon-γ is an antitumor immunological cytokine that is elevated during tumor cell death. The staining shows that IFN-γ secretion increases with IL-1RA treatment (left panel), suggesting that there is increased tumor cell death in the IL-1RA treated organoid (Figure 5).

### 3.2. Human PDAC Organoids

**Figure 6:**
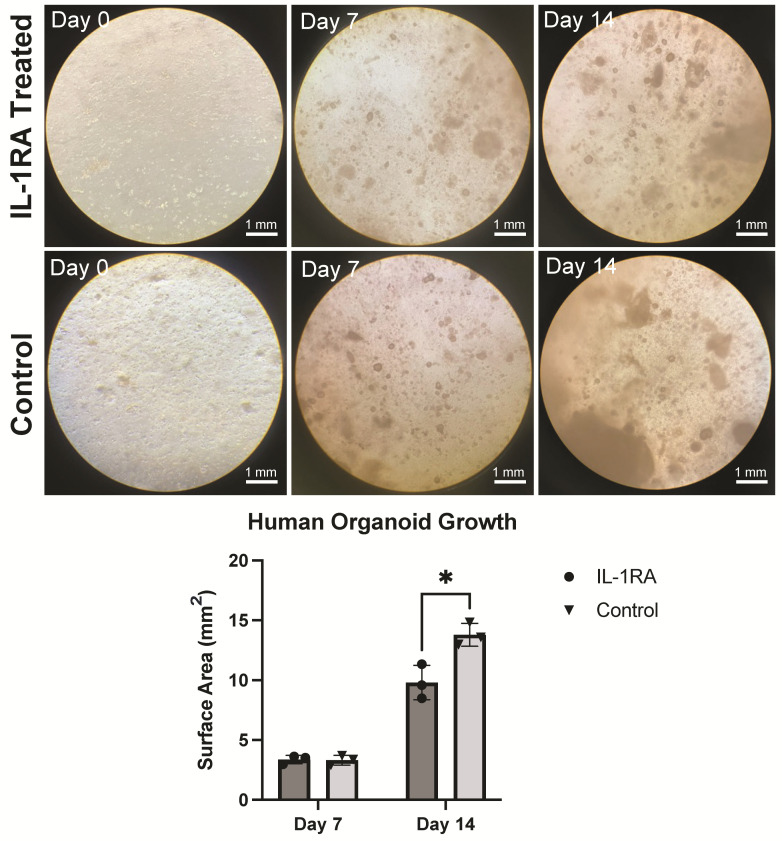
Human pancreatic ductal adenocarcinoma (PDAC) tumor organoid that has been implanted in the collagen gel at post-implantation days 0, 7, and 14. The untreated control tumor organoid (**bottom row**) is compared with the IL-1 receptor antagonist (IL-1RA)-treated tumor organoid (**top row**). Treatment was administered starting on day 7. On day 7, the organoids are approximately the same size; by day 14, the control untreated organoid is covering significantly more surface area than the IL-1RA-treated organoid (*p* < 0.05). The single asterisk (*) indicates that the *p* value is less than or equal to 0.05, but greater than 0.01.

We then wanted to test whether this technique could be used to generate organoids from human PDAC tumor tissue. Tumor fragments were seeded in the collagen gel matrix and allowed to proliferate for 7 days before treatment. By day 7 we could see the formation of small spheroid structures. By day 14 these structures had expanded and aggregated into larger tumor-like structures (Figure 6).

**Figure 7:**
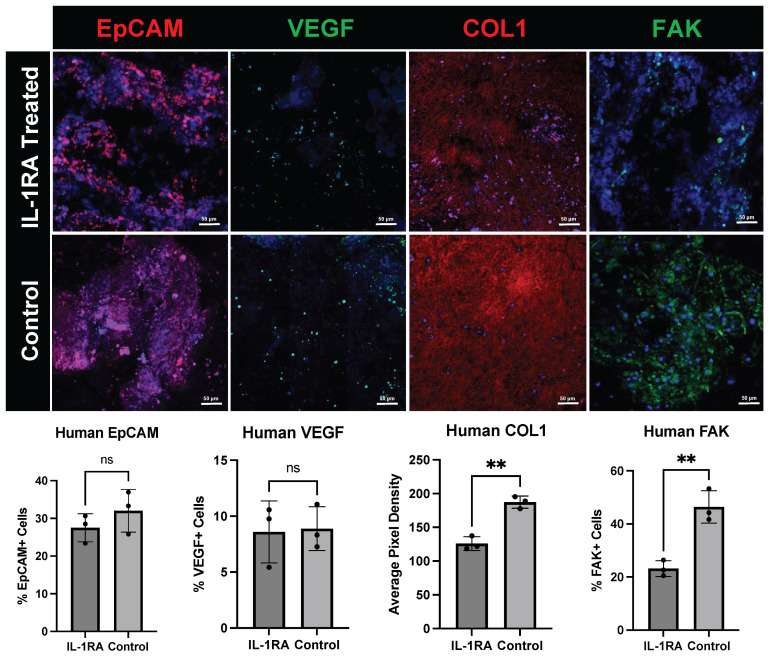
Immunofluorescence staining of human pancreatic ductal adenocarcinoma (PDAC) organoids harvested on day 14. The tumor organoids were either untreated control (**bottom row**) or treated with IL-1 receptor antagonist (IL-1RA) (**top row**). IL-1RA treatment slightly decreases but does not significantly alter the expression pattern of epithelial cell adhesion molecule (EpCAM), a tumor cell marker. Vascular endothelial growth factor (VEGF), a regulator of angiogenesis, also remained unchanged with treatment. ‘ns’ indicates that the difference between treatment groups was not significant. IL-1RA treatment also decreases the presence of certain cancer-associated fibroblast (CAF) subpopulations. Following IL-1RA treatment, we see a significant decrease in the expression of collagen 1 (COL1), a protein secreted by myCAFs (*p* < 0.005) and focal adhesion kinase (FAK), a marker of cell adhesion and migration. (*p* < 0.005). Two asterisks (**) indicate that the *p* value is less than or equal to 0.01, but greater than 0.001.

Immunofluorescence staining of the human PDAC organoids harvested on day 14 (Figure 7) demonstrates that the relevant cell types within the TME are preserved in culture. EpCAM staining of epithelial tumor cells shows a change in tumor morphology in the IL-1RA-treated group compared to the control group. The expression of vascular endothelial growth factor (VEGF), a key regulator of angiogenesis, is relatively unchanged with IL-1RA treatment. The expression of collagen 1 (COL1), a structural ECM protein and marker of myCAFs, was significantly decreased in PDAC organoids treated with IL-1RA. Expression of Focal Adhesion Kinase (FAK), a tyrosine kinase responsible for regulating cell migration, cell adhesion signaling, and mechanosensing, was also significantly decreased with IL-1RA treatment. FAK is expressed by many cell types including tumor cells, endothelial cells, and CAFs.

**Figure 8:**
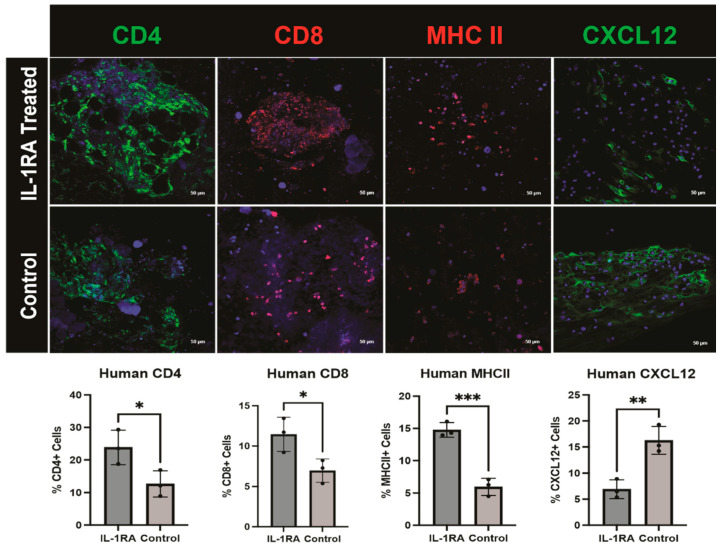
Immunofluorescence staining of human pancreatic ductal adenocarcinoma (PDAC) organoids harvested on day 14. The tumor organoids were either untreated control (**bottom row**) or treated with IL-1 receptor antagonist (IL-1RA) (**top row**). There was significantly increased expression of CD4 (*p* < 0.05) and CD8 (*p* < 0.05) immune cell markers following treatment. The single asterisk (*) indicates that the *p* value is less than or equal to 0.05, but greater than 0.01. Major histocompatibility complex II (MHCII), a macrophage and antigen-presenting CAF (apCAF) marker, is significantly increased with IL-1RA treatment (*p* < 0.001). Three asterisks (***) indicate that the *p* value is less than or equal to 0.001, but greater than 0.0001. IL-1RA treatment significantly decreases C-X-C motif chemokine ligand 12 (CXCL12), a marker of immunomodulatory CAFs (iCAFs) (*p* < 0.01). Two asterisks (**) indicate that the *p* value is less than or equal to 0.01, but greater than 0.001.

Levels of CD4+ and CD8+ immune cells were significantly elevated in the tumor organoids treated with IL-1RA. Major histocompatibility complex II (MHCII), a marker of macrophages and antigen-presenting CAFs (apCAFs), another CAF subtype involved in immune recognition and T cell activation [1], increases significantly with IL-1RA treatment. We see a significant decrease in the expression of the immunomodulatory CAF (iCAF) marker C-X-C motif chemokine ligand 12 (CXCL12) following IL-1RA treatment (Figure 8). iCAFs are characterized by their inflammatory phenotype and expression of inflammatory cytokines like CXCL12, Interleukin-6 (IL-6), and Interleukin-1 (IL-1) [1,21]. These inflammatory factors can enhance tumor cell proliferation, survival, and metastasis, while also mediating drug resistance and diminishing the immune system’s capacity to suppress tumor growth [1,21]. These combined findings are consistent with anti-fibrotic effects and immune-cell-mediated tumor cell killing.

## 4. Discussion

We chose to culture the PDAC organoids using an ALI model because it enables us to more accurately recapitulate the genetic and morphological properties of the original tumor [26]. This method allows us to preserve all cell types within the TME, including epithelial tumor cells, endothelial cells, immune cells, cancer-associated fibroblasts, and other stromal cells [32]. We have demonstrated that the complete reconstruction of the TME is possible by using the ALI culturing method. Other organoid culture methods, such as submerged Matrigel culture, 2D culture, and microfluidic 3D culture, have limitations that ALI organoids do not [33]. Matrigel cultures lack native immune and stromal cells; they must be supplemented and co-cultured [26]. Microfluidic 3D culture is more suitable than submerged Matrigel culture but still has limitations, requiring specialized equipment and is only able to sustain a very small number of cells [26]. Two-dimensional-cultured cells are unable to mimic the natural structures of tissues or tumors, and interactions between cells and the extracellular environment are not representative of the TME [33]. These culture methods are not suitable for the purpose of patient-derived organoids and the development of precision medicine treatments.

We selected days 7 and 14 as the timepoints of interest in this study. By day 7, the tumor organoid is established, proliferating, and ready to begin treatment. Around day 14, the organoids are confluent and ready to passage. Organoids can survive in culture for much longer than 14 days. With proper maintenance they will survive for many months, even exceeding one year. Studies have shown that there is little change between early- and late-passage cancer organoids [34]. Unlike other organoids, cancer organoids exhibit irregular morphologies that recapitulate the properties of the original tumor [26,34]. However, without supplementation, the populations of native tumor immune cells do not survive for more than a few days. With cytokine supplementation, immune-organoid cultures can be maintained for at least 14 days [35]. We were interested in the immune interactions that occurred following IL-1RA treatment and felt it best to analyze these interactions while most native immune cells were still viable. It is unclear what the effects of IL-1RA treatment on the organoids would yield after day 14. IL-1 plays a multifaceted role in the TME. It is produced by various cells and influences both tumor progression and antitumor immunity [36]. In future studies the effects of IL-1RA treatment on ‘long-term’ organoids should be investigated.

When utilizing patient-derived organoids for the purpose of developing personalized treatments, one needs to be able to effectively generate and test the organoids in a short period of time, which we have achieved in this study. Several recent studies have shown that the results of chemotherapy and immunotherapy treatment in organoids correlate very well with results in patients, making this patient-derived organoid strategy very exciting [15]. Currently, precision medicine antitumor therapy tested in organoids is not widely performed, but the literature [15] suggests that it will soon become a mainstream practice. To study the effects of treatment on patient-derived organoids, we believe that the ALI culture method is optimal due to its ability to conserve the cell types and features of the tumor microenvironment [6,37].

Recent studies have focused on cellular immunotherapy to treat tumors rather than chemotherapy [5,15]. Genetic and histological analysis of PDAC suggest that myCAFs and iCAFs both have an immunosuppressive effect on the tumor [17,38]. MyCAFs form a fibrotic barrier of extracellular matrix proteins, like collagen, around the tumor known as desmoplasia. Desmoplasia is hypothesized to inhibit immune cells and chemotherapy from entering the tumor. In this study we found that COL1 expression is in human PDAC organoids decreased with IL-1RA treatment, suggesting decreased CAF-induced collagen deposition and improved access to the tumor. Cell adhesion and migration marker FAK is also significantly reduced following IL-1RA treatment, possibly resulting in decreased tumorigenesis, decreased angiogenesis, and increased immune recognition. iCAFs are associated with poor prognosis as they inhibit immune recognition of the tumor [39]. Recent studies show that the iCAF marker CXCL12 is greatly reduced by IL-1RA treatment [40], which we were able to replicate and observe in this study. Decreasing the iCAF population should make the PDAC tumor more recognizable by the immune system. Consistent with enhanced immune recognition is the fact that the intra-tumoral expression of CD4+ and CD8+ immune cells is increased following IL-1RA treatment. Furthermore, macrophages and antigen-presenting CAFs expressing MHCII are also increased with IL-1RA treatment [41]. Decreased expression of myCAF and iCAF markers and increased expression of immune and apCAF markers suggest decreased fibrosis and increased immune recognition and immune-mediated tumor cell death in the PDAC organoids. This is further supported by the presence of interferon-γ in the mouse PDAC tumor organoids. The mechanism of immune cell killing of tumor is substantiated by alterations in epithelial tumor cell morphology and increased levels of IFN-γ. Combining effective anti-CAF treatments with established antitumor agents is a promising strategy for the treatment of solid tumors like PDAC.

These findings suggest that the tumor microenvironment recapitulated by the ALI method of organoid culture demonstrates a complete tumor microenvironment. The findings following IL-1RA treatment of mouse and human PDAC organoids result in decreased fibrosis, enhanced immune tumor recognition, and an enhanced antitumor effect. Other groups have performed studies in colorectal cancer organoids with treatments including chemoradiation therapy, which is also able to demonstrate significant antitumor results [20]. These preliminary studies support the use of precision anticancer therapy and patient derived organoids. In future studies, finding the right combination of anti-CAF therapies, immunotherapies, and established antitumor agents like chemotherapy and radiation may result in treatment methods that yield complete responses in both cancer organoids and patients.

## 5. Conclusions

This study highlights the effectiveness of the ALI culturing method in generating patient-derived cancer organoids. These organoids can replicate the morphological and genetic qualities of the tumor microenvironment and provide a valuable platform for testing cancer treatments. The results also emphasize the promising role of IL-1RA in PDAC as an anti-CAF therapy, improving immune recognition and enhancing anti-tumor effects. These findings support the integration of organoid-based models into cancer research and treatment, offering a potential breakthrough in the development of more-targeted, effective therapies for solid tumors like pancreatic cancer.

## Data Availability

Data are contained within the article.

## Abbreviations

The following abbreviations are used in this manuscript:

PDAC	Pancreatic ductal adenocarcinoma
TME	Tumor microenvironment
IL-1RA	Interleukin 1 receptor antagonist
CAF	Cancer-associated fibroblast
myCAF	Myofibrotic cancer-associated fibroblast
iCAF	Immunomodulatory cancer-associated fibroblast
apCAF	Antigen-presenting cancer-associated fibroblast
PDO	Patient-derived organoid
αSMA	Alpha smooth muscle actin
COL1	Collagen 1
FAK	Focal adhesion kinase
EpCAM	Epithelial cell adhesion molecule
CK19	Cytokeratin 19
CXCL12	C-X-C motif chemokine ligand 12
MHC II	Major histocompatibility complex II
VEGF	Vascular endothelial growth factor
