# Correction: Morgan et al. Precision Medicine: IL-1RA and Pancreatic Cancer Organoids. *Biology* 2025, *14*, 604

**DOI:** 10.3390/biology14091133

**Published:** 2025-08-27

**Authors:** Annah G. Morgan, Michelle F. Griffin, Michael T. Longaker, Jeffrey A. Norton

**Affiliations:** 1 Hagey Laboratory for Pediatric Regenerative Medicine, Division of Plastic and Reconstructive Surgery, Department of Surgery, Stanford University School of Medicine, Stanford, CA 94305, USA; agmorgan@stanford.edu (A.G.M.); mgriff12@stanford.edu (M.F.G.); longaker@stanford.edu (M.T.L.); 2 Division of Plastic and Reconstructive Surgery, Department of Surgery, Stanford University School of Medicine, Stanford, CA 94305, USA; 3 Division of General Surgery, Department of Surgery, Stanford University School of Medicine, Stanford, CA 94305, USA

## 1. Error in Figure

In the original publication [1], there were mistakes in Figures 4 and 8 as published.

### 1.1. Figure 4

In the original publication, there was a mistake in Figure 4 as published. The incorrect image for ‘αSMA, IL-1RA treated’ was included due to unclear labeling of the image file. The corrected Figure 4 appears below.

### 1.2. Figure 8

In the original publication, there was a mistake in Figure 8 as published. The incorrect image for ‘CD8, IL-1RA treated’ was included due to unclear labeling of the image file. The corrected Figure 8 appears below.

The authors state that the scientific conclusions are unaffected.

This correction was approved by the Academic Editor. The original publication has also been updated.

## Figures and Tables

**Figure 4:**
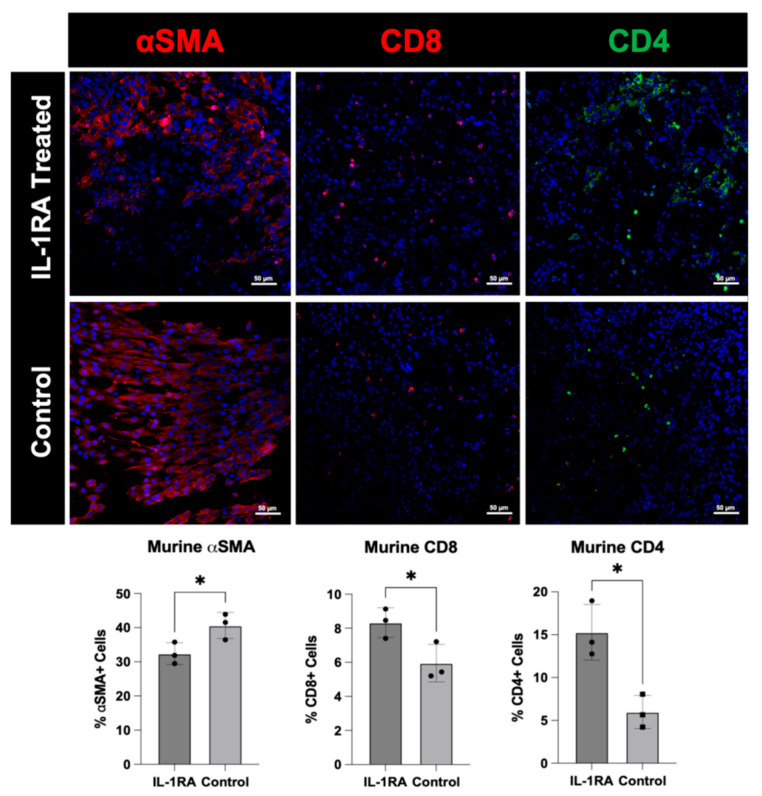
Immunofluorescence staining of murine pancreatic ductal adenocarcinoma (PDAC) organoids harvested on day 14. The tumor organoids were either untreated control (**bottom row**) or treated with IL-1RA (**top row**). IL-1RA treatment significantly decreased expression of alpha-smooth muscle actin (α-SMA) (*p* < 0.05), and significantly increased the expression of CD4+ (*p* < 0.05) and CD8+ (*p* < 0.05) immune cells. The single asterisk (*) indicates that the *p* value is less than or equal to 0.05, but greater than 0.01.

**Figure 8:**
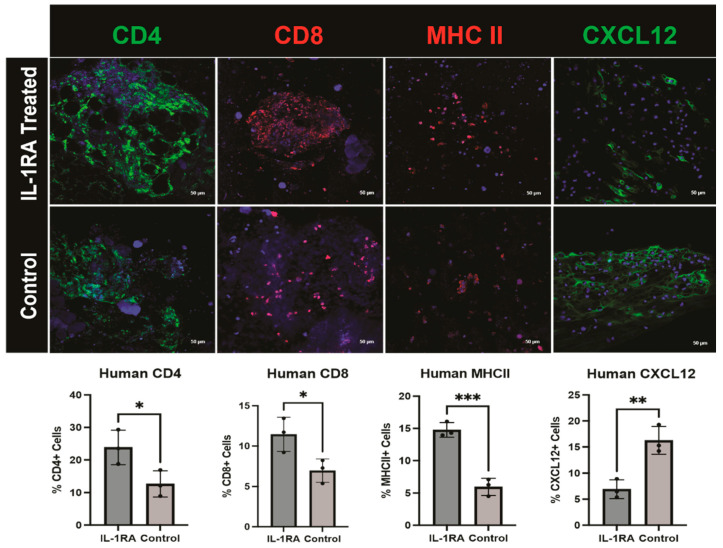
Immunofluorescence staining of human pancreatic ductal adenocarcinoma (PDAC) organoids harvested on day 14. The tumor organoids were either untreated control (**bottom row**) or treated with IL-1 receptor antagonist (IL-1RA) (**top row**). There was significantly increased expression of CD4 (*p* < 0.05) and CD8 (*p* < 0.05) immune cell markers following treatment. The single asterisk (*) indicates that the *p* value is less than or equal to 0.05, but greater than 0.01. Major histocompatibility complex II (MHCII), a macrophage and antigen-presenting CAF (apCAF) marker, is significantly increased with IL-1RA treatment (*p* < 0.001). Three asterisks (***) indicate that the *p* value is less than or equal to 0.001, but greater than 0.0001. IL-1RA treatment significantly decreases C-X-C motif chemokine ligand 12 (CXCL12), a marker of immunomodulatory CAFs (iCAFs) (*p* < 0.01). Two asterisks (**) indicate that the *p* value is less than or equal to 0.01, but greater than 0.001.
